# An important role of cutaneous lymphatic vessels in coordinating and promoting anagen hair follicle growth

**DOI:** 10.1371/journal.pone.0220341

**Published:** 2019-07-25

**Authors:** Sun-Young Yoon, Lothar C. Dieterich, Sinem Karaman, Steven T. Proulx, Samia B. Bachmann, Carol Sciaroni, Michael Detmar

**Affiliations:** 1 Institute of Pharmaceutical Sciences, Swiss Federal Institute of Technology, ETH Zurich, Zurich, Switzerland; 2 School of Pharmacy, Sungkyunkwan University, Suwon, Republic of Korea; Yale University School of Medicine, UNITED STATES

## Abstract

The lymphatic vascular system plays important roles in the control of tissue fluid homeostasis and immune responses. While VEGF-A-induced angiogenesis promotes hair follicle (HF) growth, the potential role of lymphatic vessels (LVs) in HF cycling has remained unknown. In this study, we found that LVs are localized in close proximity to the HF bulge area throughout the postnatal and depilation-induced hair cycle in mice and that a network of LVs directly connects the individual HFs. Increased LV density in the skin of K14-VEGF-C transgenic mice was associated with prolongation of anagen HF growth. Conversely, HF entry into the catagen phase was accelerated in K14-sVEGFR3 transgenic mice that lack cutaneous LVs. Importantly, repeated intradermal injections of VEGF-C promoted hair growth in mice. Conditioned media from lymphatic endothelial cells promoted human dermal papilla cell (DPC) growth and expression of IGF-1 and alkaline phosphatase, both activators of DPCs. Our results reveal an unexpected role of LVs in coordinating and promoting HF growth and identify potential new therapeutic strategies for hair loss-associated conditions.

## Introduction

The hair follicle (HF) is a mini-organ of the skin that continuously cycles through rapid growth (anagen phase), apoptosis-driven regression (catagen phase) and relative quiescence (telogen phase) [1]. The HF-associated blood vascular system undergoes a massive expansion during the anagen phase and a rapid involution during the catagen regression phase [2, 3]. In previous studies, we found that the production of vascular endothelial growth factor (VEGF)-A is up-regulated during the anagen phase and that transgenic mice with increased levels of cutaneous VEGF-A have larger HFs than their wildtype littermates [4]. Conversely, blockade of VEGF-A resulted in thinner HFs [5] and hair thinning has been reported under anti-angiogenic therapy [6]. Indeed, it has been proposed that stimulation of blood vessels by induction of VEGF-A production represents a major mechanism of action of the hair growth-promoting drug minoxidil [7].

While blood vessels supply peripheral tissues with oxygen and nutrients and are associated with leakage of fluid into these tissues, a major function of lymphatic vessels (LVs) is the drainage of tissue fluid and cells from the periphery and their recirculation, via collecting LVs and the thoracic duct, to the blood vascular system [8–11]. Beyond fluid drainage, LVs play important roles in the transport of dendritic and other immune cells to the lymph nodes, and recent evidence indicates that they might also be involved in the control of autoimmunity to peripheral tissue antigens [12, 13]. Impairment of lymphatic function often leads to edema, impaired immunity, impaired wound healing and tissue fibrosis [14]. There has been a strongly elevated interest in lymphatic research since an involvement of LVs in the pathogenesis of an increasing number of diseases has been found during the last few years, including metastatic cancer, chronic autoimmune diseases, impaired wound healing, organ transplant rejection, myocardial infarction and atherosclerosis [15–25].

While studying the inflammatory response in transgenic mice with skin-specific overexpression of the lymphangiogenic growth factor VEGF-C (K14-VEGF-C) [26], we observed that hair regrowth after skin shaving was accelerated in these mice (unpublished observation). We therefore wondered whether LVs might play an active role in the homeostasis of HF cycling. To this end, we first characterized the lymphatic vascularity during postnatal HF cycling in mice. We then investigated genetic mouse models with enhanced or decreased lymphatic vascularization of the skin: K14-VEGF-C transgenic mice that have an increased cutaneous lymphatic network [27], and transgenic mice with skin-specific overexpression of a soluble, extracellular domain of VEGF receptor-3 (K14-sVEGFR3 transgenic mice) [28]. The soluble VEGFR-3 scavenges its ligands VEGF-C and VEGF-D and thereby prevents these ligands from activating VEGFR-3 on the lymphatic endothelium, resulting in the absence of LVs in the skin [28]. We also investigated the biological effects of intradermal VEGF-C protein delivery on hair growth. Our results reveal an unexpected, major role of lymphatic endothelium in promoting anagen HF growth in mice.

## Materials and methods

### Mouse models

K14-VEGF-C transgenic mice on the FVB background that express VEGF-C under control of the keratin 14 (K14) promoter, and K14-sVEGFR3 transgenic mice that express a sVEGFR3 [27, 28] were kindly provided by Dr. Kari Alitalo, University of Helsinki. To investigate the effects of lymphatic vasculature on the hair cycle after depilation-induced hair regeneration, the back skin of 8-week-old female K14-VEGF-C or K14-sVEGFR3 transgenic mice in the telogen phase was depilated using wax as described [1, 29], resulting in the synchronized induction of new anagen follicle growth. The number of mice (WT: n = 24, K14-VEGF-C transgenic: n = 10, K14-sVEGFR3 transgenic mice: n = 8) used for each experiment is indicated in the figure legends. Mice were sacrificed with an overdose of anaesthesia (160 mg kg^−1^ ketamine; 0.4 mg kg^−1^ medetomidine) at days 15 and 18 after depilation, and the back skin was taken for histological analysis. Grading the hair cycle phases was done using hematoxylin and eosin (H&E)-stained paraffin sections, according to established guidelines [1]. To measure the bulb diameter, 3 images/mouse were acquired and the bulb diameter was measured at the level of the largest diameter (“Auber’s line”) of the hair bulbs with a clearly visible dermal papilla (DP) [4]. For quantitative analysis, the Image J software (National Institutes of Health, Bethesda, MD, USA) was used. To examine the effects of the lymphatic vasculature on the postnatal hair cycle, back skin samples were obtained from WT (n = 4) and K14-VEGF-C transgenic female mice (n = 9) at postnatal days 25.

To investigate the effects of recombinant VEGF-C on HF cycling, anagen induction was performed in 8-week-old female C57BL/6J mice (Janvier Labs, Le Genest-Saint-Isle, France) as described previously [30]. The back skin of mice in the telogen phase was shaved with a clipper. Vehicle (PBS containing 0.1% bovine serum albumin [BSA]) alone (n = 4) or vehicle containing VEGF-C (R&D Systems, Minneapolis, MN, USA; 200 ng in 20 μL, n = 5) or minoxidil (MNX; Sigma-Aldrich, St. Louis, MO, USA, positive control; 20 μg in 20 μL, n = 5) was applied via intradermal injection every day for 40 days and back skin samples were obtained at the site of intradermal injection and processed for H&E-stained paraffin sections. Prox1-tdTomato reporter mice (FVB background) that express tdTomato under the Prox1 promoter (Mutant Mouse Resource & Research Centers supported by NIH, stock number: 036531-UCD) were used to analyze the dermal lymphatic network. Unfixed back skin samples were imaged using a Zeiss Axiozoom V16 fluorescence stereomicroscope. All experimental procedures were conducted according to animal protocols approved by the Kantonales Veterinaeramt Zuerich (protocol 237/2013).

### Immunofluorescence stainings

Back skin samples were embedded in OCT (Leica Biosystems, Newcastle, UK) and frozen in liquid nitrogen. 10-μm frozen sections were fixed in 4% paraformaldehyde (PFA) for 15 min at room temperature (RT), washed in PBS and incubated with blocking solution (5% donkey serum, 0.2% BSA and 0.3% Triton X-100 in PBS) for 1 h at RT. Next, the sections were stained with primary antibodies overnight at 4°C and, after several washes, incubated with secondary antibodies for 30 min at RT. Primary antibodies were as follows: rat anti-CD31 (BD Biosciences, San Jose, CA, USA), rabbit anti-LYVE-1 (Angiobio, Del Mar, CA, USA), goat anti-LYVE-1 (R&D Systems), rat anti-CD68 (Abcam, Cambridge, MA, USA), goat anti-podoplanin (R&D Systems), rabbit anti-cytokeratin 15 (Abcam), rat anti-Foxp3 (eBioscience, San Diego, CA, USA), and goat anti-Prox1 (R&D systems). Secondary antibodies (all from Thermo Fisher, San Jose, CA, USA) were as follows: donkey anti-rabbit Alexa Fluor 488, 594 or 647, donkey anti-rat Alexa Fluor 488 or 594, donkey anti-goat Alexa Fluor 488 or 594, and chicken anti-goat Alexa Fluor 647. Hoechst 33342 (Invitrogen, Carlsbad, CA, USA) was used for nuclear staining. Immunofluorescence images were acquired by an Axioskop 2 mot plus microscope (Carl Zeiss, Jena, Germany) and Z stacks of images were obtained using a Zeiss LSM 710 FCS confocal microscope.

### Cell culture

The methods used for culturing human DPCs have been described previously [31, 32]. DPCs (ScienCell Research Laboratories, Carlsbad, CA, USA) were cultured in Dulbecco’s modified Eagle’s medium (DMEM; Gibco, Grand Island, NY, USA) supplemented with 10% fetal bovine serum (FBS; Gibco), 10 ng/mL basic fibroblast growth factor (Peprotech, London, UK) and antibiotic/antimycotic solution (Gibco). Primary human LECs and BECs isolated from foreskin were cultured as described previously [33]. LECs were cultured on 10 μg/mL fibronectin (Millipore, Billerica, MA, USA)-coated dishes in endothelial cell basal medium (EBM; Lonza, Walkersville, MD, USA), supplemented with 20% FBS, 2 mM l-glutamine (Gibco), antibiotic-antimycotic solution, 10 μg/mL hydrocortisone (Sigma-Aldrich) and 25 μg/mL cAMP (Sigma-Aldrich). BECs were cultured on 10 μg/mL fibronectin-coated dishes in EBM supplemented with 20% FBS, 2 mM l-glutamine, 1x antibiotic-antimycotic solution and 0.4% endothelial cell growth supplement (ECGS; PromoCell). Human dermal fibroblasts (DFs) were cultured in DMEM supplemented with 10% FBS and antibiotic/ antimycotic solution. Cells were incubated at 37°C in a 5% CO_2_ incubator.

### Conditioned media

To investigate the effects of conditioned media (CM) on DPCs, CM of LECs, BECs and DFs were collected. When cells reached 80% confluence, the growth medium was replaced with EBM containing 2% FBS for LECs and BECs or with DMEM containing 2% FBS for DFs. CM were collected after 48 h and centrifuged. For the preparation of control CM, DMEM or EBM containing 2% FBS was added to empty cell culture dishes and collected after 48 h. DPCs were incubated with 10, 30, 50 or 90% LEC-CM, BEC-CM or DF-CM for 72 h or 100 ng/ml IGF-1 as a positive control. For the quantification of DPC proliferation, Cell Counting Kit-8 (Sigma-Aldrich) was used according to the manufacturer’s instructions. After 72 h, the absorbance at 450 nm was measured using a microplate reader (Spectramax Gemini EM, Molecular Devices, Sunnyvale, CA, USA).

### Quantitative real time-polymerase chain reaction (qRT-PCR)

Total RNA was isolated from DPCs using NucleoSpin RNA (Macherey-Nagel, Düren, Germany) and 1 μg of total RNA was used for the cDNA synthesis reaction using the High Capacity Reverse Transcription kit (Applied Biosystems, Foster City, CA, USA). PCR was performed on a 7900HT Fast Real-Time PCR System (Applied Bio-systems) using FastStart SYBR green master mix (Roche Diagnostics, Basel, Switzerland) according to the manufacturer’s instructions. Gene expression was normalized to the control gene Rplp0 (36B4). Primer information is provided in S1 Table. All experiments were independently repeated at least 3 times.

### Western blotting

Total protein from DPCs was extracted in a buffer containing 25 mM HEPES, 150 mM NaCl, 1% Triton X-100, 10% glycerol, 5 mM EDTA, 10 mM NaF, 2 mM Na_3_VO_4_, and protease inhibitor cocktail (Roche Diagnostics). Proteins were separated using 10% sodium dodecyl sulfate-polyacrylamide gel electrophoresis and transferred to a polyvinylidene fluoride membrane (EMD Millipore, Billerica, MA, USA) using a wet transfer system. The blotted membranes were incubated with primary antibodies at 4°C. The following antibodies were used: anti-total Akt, anti-phosphorylated Akt (Cell Signaling Technology, Beverly, MA, USA) and anti-beta-actin (Abcam). Membranes were then probed with an anti-rabbit-IgG-horseradish peroxidase conjugate (GE Healthcare, Hilleroed, Denmark). Antibody–antigen complexes were detected using the ECL system (Thermo Scientific).

### Statistical analyses

The statistical tests used are indicated in the figure legends and results were considered significant at *P*<0.05. The diameters of hair bulbs were analyzed with the two-tailed unpaired t-test for each time point. For the studies with CM, mRNA expression levels were analyzed using the two-tailed paired t-test (GraphPad Prism, version 5.0a, San Diego, CA, USA). The differences of HF cyling between WT and transgenic mice were compared by Fisher’s exact test, calculated online at QuickCalcs, GraphPad Software.

## Results

### LVs are localized in close proximity to the bulge and the bulb region of murine HFs

**Fig 1:**
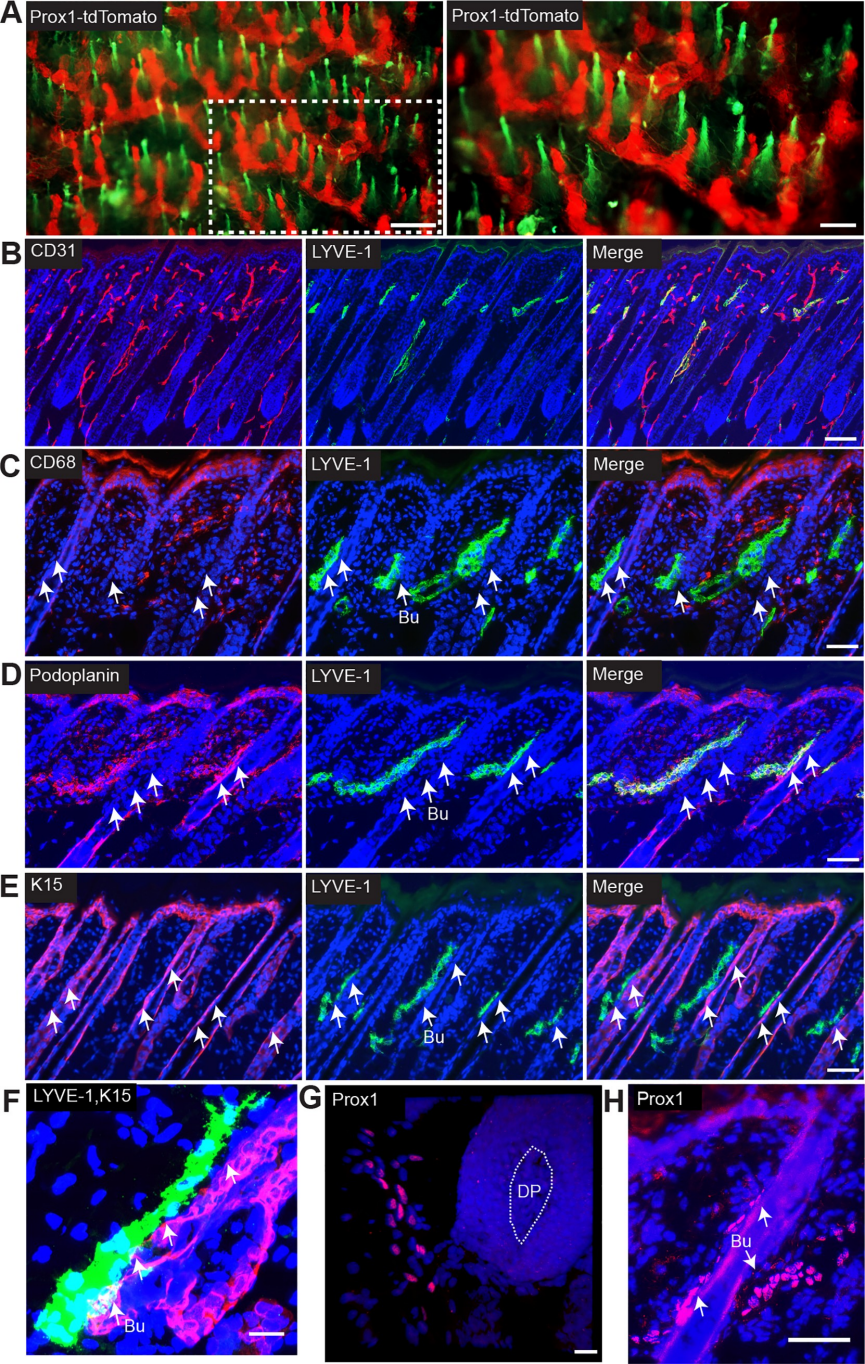
Lymphatic vessels are localized in close proximity to the HF. (A) Representative image of the back skin of a Prox1-tdTomato mouse (red, lymphatic vasculature). Autofluorescence from the hair fiber (green). The dotted area on the left is magnified in the right panel. Scale bars: 200 μm (left panel), 100 μm (right panel). (B-F) Immunofluorescent staining of 10-μm frozen sections of back skin (anagen phase, postnatal day 8) for CD31 (B; panendothelial marker, red), LYVE-1 (B-F; lymphatic marker, green), CD68 (C; macrophage marker, red), podoplanin (D; lymphatic marker, red) and cytokeratin 15 (E and F; marker of HF stem cells, red). White arrows indicate the bulge area (Bu). (G) Staining of 50-μm cryosections for Prox1 (lymphatic-specific transcription factor, red). Maximum intensity projection of a Z stacks of images acquired using a Zeiss LSM 710 confocal microscope. (H) Immunofluorescent staining of back skin (anagen phase, postnatal day 8) for Prox1 (red). (B-H) Nuclear staining with Hoechst 33342 (blue). Scale bars: 100 μm (B); 50 μm (C-E, H); 20 μm (F and G). DP = dermal papilla, Bu = bulge area.

To characterize the dermal lymphatic vascularity, we investigated the LV distribution in the back skin of Prox1 (lymphatic-specific transcription factor)-tdTomato reporter mice that express tdTomato under control of the Prox1 promoter. By fluorescence stereomicroscopy, we found that a network of LVs directly connects the individual HFs (Fig 1A). To further characterize the location and morphology of LVs during the hair cycle, back skin samples of wildtype mice in the anagen phase (postnatal day 8) were stained for the panvascular marker CD31 and the lymphatic vessel marker LYVE-1. CD31^+^/LYVE-1^+^ LVs were located in close proximity to anagen HFs, predominantly in an area close to the hair bulge (Fig 1B). Since LYVE-1 may also be expressed by some cutaneous macrophages, we performed double immunofluorescence stainings to confirm the identity of HF-associated LVs. The LYVE-1-positive vessels did not express the macrophage marker CD68 (Fig 1C) but co-expressed the LV marker podoplanin (Fig 1D). To further characterize the location of LVs in relation to the HF, we performed double immunofluorescence stainings for keratin 15, a putative marker of follicular stem cells, and LYVE-1. LVs were frequently localized near the stem cell region of the bulge of the HF (Fig 1E) and appeared to be directly attached to the bulge region (Fig 1F). This finding was confirmed by tissue whole-mount stainings for LYVE-1 and keratin 15, followed by light-sheet microscopy, revealing precollecting vessels that extended along HFs during the anagen phase (S1 Video) but not during the telogen phase (S2 Video). The LVs in close proximity to the hair bulbs (Fig 1G) and the hair bulge (Fig 1H) also expressed the lymphatic-specific transcription factor Prox1^+^.

### Prolonged depilation-induced anagen hair growth in K14-VEGF-C transgenic mice

**Fig 2:**
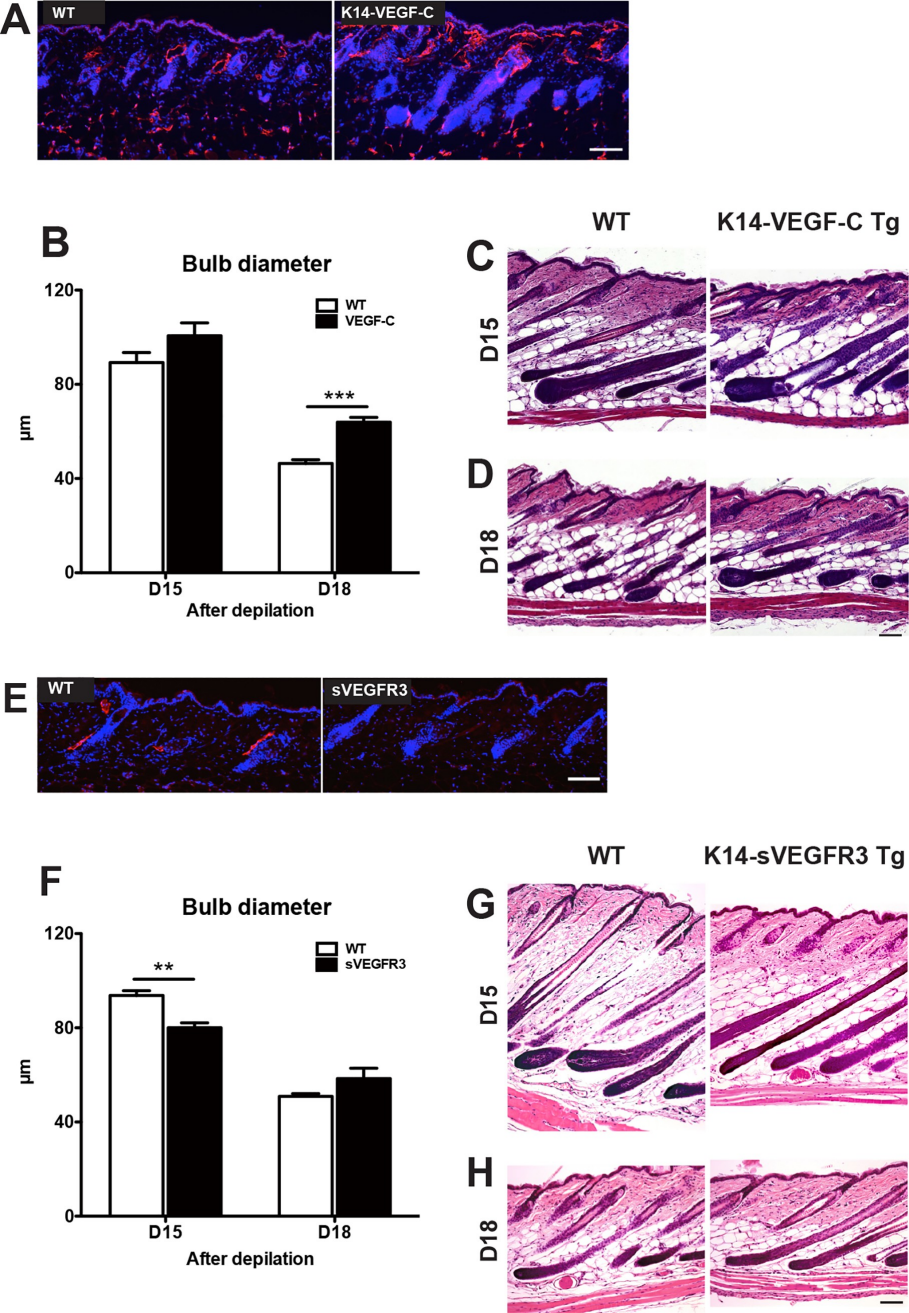
Prolongation of anagen HF growth in K14-VEGF-C transgenic mice and early entry into the catagen phase in K14-sVEGFR3-Ig transgenic mice. (A and E) Immunofluorescent staining of 10-μm frozen sections of back skin (anagen phase, postnatal day 8) for LYVE-1 (lymphatic marker, red). Nuclear staining with Hoechst 33342 (blue). (B and F) In H&E stained paraffin sections, 3 images/mouse were acquired and the bulb diameter was measured at the level of the largest diameter (“Auber’s line”). Data were analyzed using the two-tailed unpaired t-test for each time point. Results are presented as mean ± standard error of the mean (SEM). ***P < 0.001, **P < 0.01 versus control group. (C-D and G-H) After depilation-induced HF regeneration, back skin samples were obtained at days 15 (C, WT: n = 6, K14-VEGF-C transgenic mice: n = 4), 18 (D, WT: n = 8, K14-VEGF-C transgenic mice: n = 6), days 15 (G, WT: n = 5, K14-sVEGFR3 transgenic mice: n = 4), 18 (H, WT: n = 5, K14-sVEGFR3 transgenic mice: n = 4). The paraffin sections were stained with H&E. Scale bar: 100 μm.

To investigate whether HF-associated LVs might have a functional role in HF growth and/or cycling, we studied depilation-induced hair regeneration in female K14-VEGF-C transgenic mice that are characterized by an increased density of LVs in the skin [27] (Fig 2A). In line with our previous findings [34, 35], we observed no differences in MECA-32^+^ blood vessels (S1 Fig). Then, we measured the diameter of the hair bulb after depilation-induced hair regeneration, which typically increases during the anagen phase, whereas it is reduced during catagen development [1]. At day 15 (late-anagen phase) after depilation, all WT and K14-VEGF-C transgenic mice were in the anagen VI phase with comparable hair bulb diameters (Fig 2B and Fig 2C). However, at day 18 (catagen phase), the diameter of the hair bulb was significantly larger in transgenic mice (Fig 2B and Fig 2D), indicating that after depilation, K14-VEGF-C transgenic mice remained longer in the anagen growth phase than WT mice during the anagen-to-catagen transition of the HF.

### Early entry into the catagen phase in K14-sVEGFR3-Ig transgenic mice

Since VEGF-C prolonged the anagen phase of the HF, we next investigated whether blockade of VEGF-C might accelerate the entry into the catagen phase. To this end, we first studied depilation-induced hair regeneration in female K14-sVEGFR3-Ig mice. These mice express a soluble form of the extracellular domain of VEGFR3, thus scavenging VEGF-C (and -D) and preventing its interaction with its receptors on lymphatic endothelial cells. Immunofluorescence stainings for LYVE-1 confirmed the absence of LVs in the back skin of K14-sVEGFR3-Ig mice, whereas WT mice showed a normal lymphatic vasculature (Fig 2E). At day 15 (late-anagen phase), only anagen VI HFs were observed in all WT mice, whereas all K14-sVEGFR3-Ig mice had already early-catagen HFs (Fig 2F). Accordingly, the thickness of the hair bulbs was significantly smaller in K14-sVEGFR3-Ig mice (Fig 2G). At day 18 (catagen phase), all mice were in mid-catagen phase (Fig 2F and Fig 2H). Taken together, these results indicate that inhibition of VEGF-C signaling in K14-sVEGFR3 transgenic mice accelerates the entry of HFs into the catagen phase during the anagen-to-catagen transition.

### VEGF-C levels are increased during the anagen phase

We next investigated whether the expression levels of VEGF-C and its receptor VEGFR3 might undergo cyclic changes during the normal postnatal hair cycle. We found that the mRNA expression levels of VEGF-C and VEGFR3 in the back skin were significantly higher during the anagen phase (at P28) than during the telogen phase (at P49) (S2A Fig). Similar results were obtained during depilation-induced HF regeneration, where the VEGF-C mRNA expression levels were significantly higher during the anagen phase (day 12 after depilation) than during the telogen phase (day 22 after depilation) (S2B Fig). By contrast, mRNA expression levels of the related lymphangiogenic growth factor VEGF-D [36] remained unchanged during postnatal and depilation-induced hair cycling (S2A Fig and S2B Fig). In addition, we measured the amount of VEGF-C secretion in conditioned media (CM) from dermal fibroblasts (DF) and dermal papilla cells (DPC) by enzyme-linked immunosorbent assay (ELISA). We found that DF-CM and DPC-CM contained VEGF-C, indicating that DF and DPC express VEGF-C (S3 Fig).

### Accelerated initiation of anagen HF growth during normal postnatal hair follicle cycling in K14-VEGF-C transgenic mice and after intradermal delivery of VEGF-C

**Fig 3:**
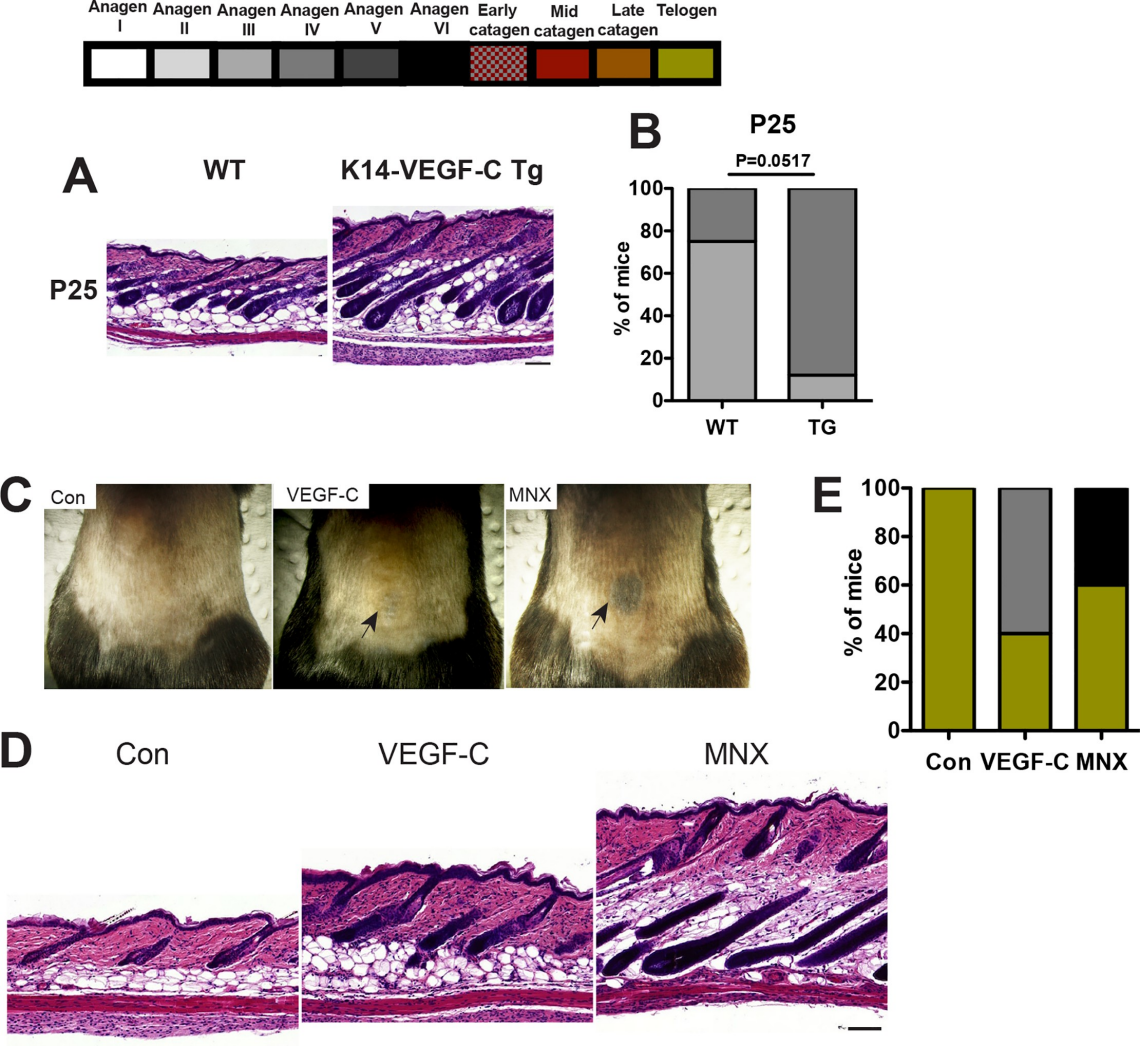
Intradermal delivery of VEGF-C promotes anagen hair growth. (A) Back skin samples were obtained at postnatal days 25 (A and B, P25, WT: n = 4, transgenic: n = 9) and paraffin sections were stained with H&E. (B) 3 images/mouse were acquired and differences in the hair cycle phase of WT and K14-VEGF-C transgenic mice were compared using Fisher’s exact test. Scale bars: 100 μm. (C) 8-week-old C57BL/6 female mice in the telogen phase were shaved on the back skin with a clipper, and were intradermally injected daily for 40 days with vehicle (n = 4), VEGF-C (n = 5) or minoxidil (MNX; n = 5). Black arrows indicate the site of intradermal injection. (D) Back skin samples were obtained at the site of intradermal injection and paraffin sections were stained with H&E. Scale bars: 100 μm. (E) Grading the hair cycle phases was performed using H&E-stained paraffin sections.

Given the cyclic changes of VEGF-C mRNA expression during the hair cycle and the prolongation of anagen HF growth in K14-VEGF-C transgenic mice, we next investigated whether cutaneous LVs might also have a biological role during normal postnatal hair cycling. At postnatal day 25 (P25; second postnatal hair cycle), 88% of female K14-VEGF-C transgenic mice were in the anagen IV phase and 12% in anagen III, whereas only 25% of WT mice were in the anagen IV phase and 75% still in anagen III (Fig 3A and Fig 3B). However, there was no differences in epidermal thickness between WT and K14-VEGF-C transgenic mice (S4 Fig). These results indicate that K14-VEGF-C transgenic mice initiated the anagen HF growth more rapidly than WT mice also during normal, postnatal HF cycling.

Next we wondered whether application of recombinant VEGF-C protein might promote anagen induction. To this end, we intradermally injected 200 ng VEGF-C, 20 μg minoxidil (positive control) or an equal volume (20 μl) PBS (negative control) into the back skin of 8-week-old female C57BL/6J mice daily for 40 days and then analyzed the hair cycle phase. After 40 days, skin pigmentation—a sign for HF entrance into the anagen phase—was observed at the site of intradermal injections in the VEGF-C- and the minoxidil-treated group, but not in the control group (Fig 3C). Analyses of histological sections revealed that 60% of the VEGF-C-treated mice and 40% of the MNX-treated mice were in the anagen hair cyle phase, whereas all vehicle-treated mice were still in the telogen phase (Fig 3D and Fig 3E). These results identify a potent activity of VEGF-C treatment in promoting HF entry into the anagen growth phase during the telogen-to-anagen transition.

### LEC conditioned media promote dermal papilla cell proliferation

**Fig 4:**
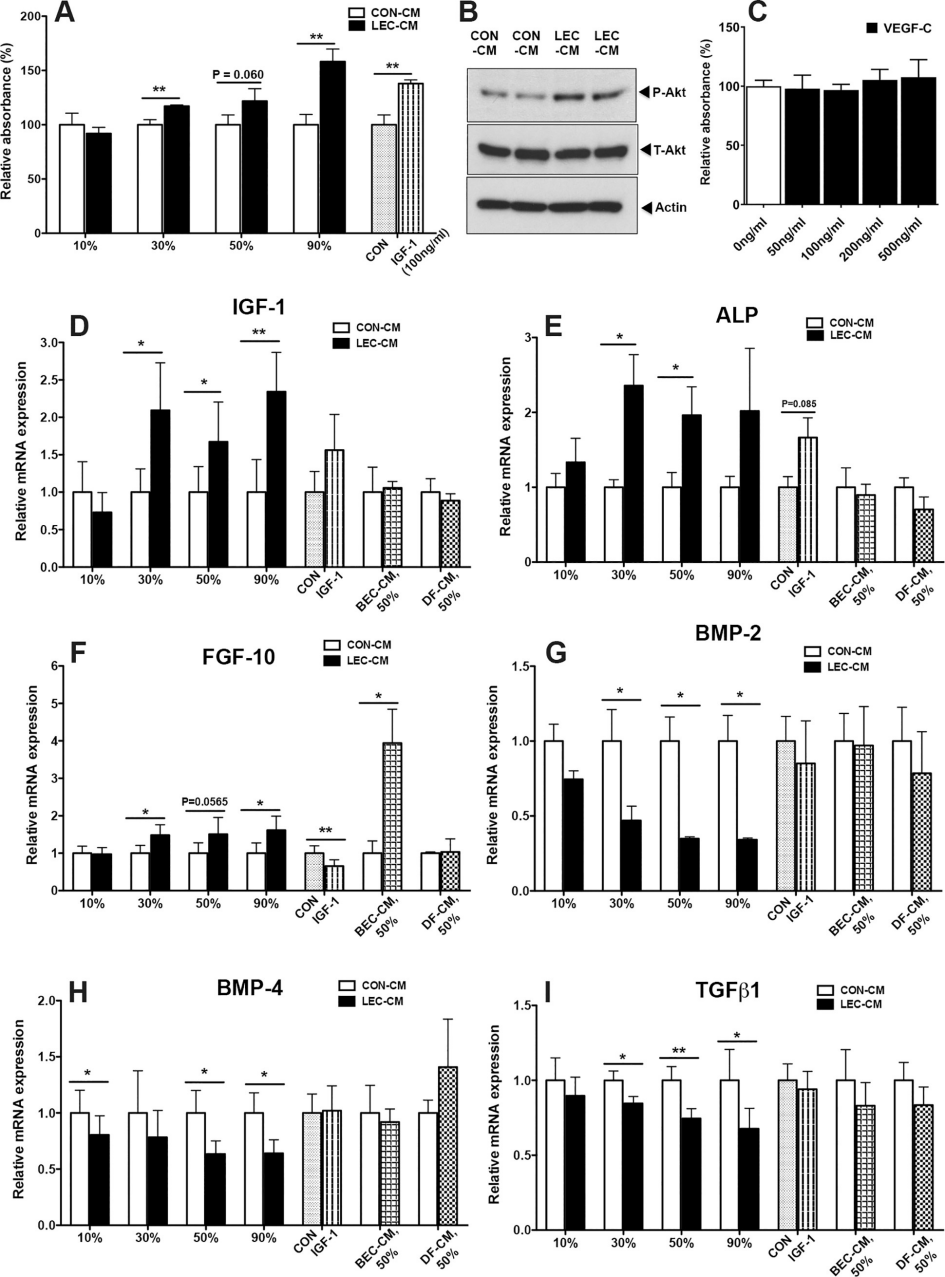
LEC conditioned media promote DPC proliferation, enhance their expression of IGF-1 and ALP, and inhibit BMP-2 and BMP-4 expression. (A) DPCs were incubated with control CM (CON-CM) or LEC-CM (10, 30, 50 or 90%), or 100 ng/ml IGF-1 as a positive control for 72 h. Cell proliferation was assessed with the CCK-8 assay. Differences in relative absorbance levels were analyzed by the two-tailed unpaired t-tests. One representative experiment is presented as mean ± standard deviation (SD). **P<0.01 versus control group. (B) DPCs were incubated with 50% LEC-CM or CON-CM for 72 h. Cells were lysed and western blotting was performed, using antibodies for total Akt (T-Akt), phosphorylated Akt (P-Akt), or beta-actin. (C) DPCs were treated with the indicated concentrations of recombinant human VEGF-C for 72 h. Cell proliferation was assessed with the CCK-8 assay. Differences in relative absorbance levels were analyzed with the two-tailed unpaired t-test. Results are presented as mean±SD. (D-I) DPCs were incubated with CON-CM or LEC-CM (10, 30, 50 or 90%), BEC-CM (50%), dermal fibroblast-CM (DF-CM; 50%), or 100 ng/ml IGF-1 for 72 h. Total RNA was isolated and qRT-PCR was performed. mRNA expression levels were analyzed using the two-tailed paired t-test. Pooled data from 4 independent experiments are presented as mean±SEM. *P<0.05, **P<0.01 versus control group.

Given the strong association between LV expansion and the prolongation of anagen HF growth in K14-VEGF-C transgenic mice, we next investigated whether lymphatic endothelial cells (LECs) might secrete paracrine factors that activate DPCs, since HF stem cells receive stimulatory signals from DPCs to drive hair cycling [37, 38]. We collected conditioned media (CM) from cultured human dermal LECs and examined their effects on cultured human DPCs. 100 ng/ml IGF-1, a growth factor known to stimulate HF growth [39], was used as a positive control. Incubation with 30%, 50% or 90% LEC-CM significantly increased DPC proliferation in a dose-dependent manner, to a similar or even higher extent than IGF-1, as compared to DPCs incubated with equivalent amounts of control conditioned media (CON-CM) (Fig 4A). In line with this, incubation of DPCs with LEC-CM also increased the phosphorylation of Akt which is thought to promote cell survival [40] (Fig 4B). On the contrary, incubation of DPCs with CM from human dermal blood vascular endothelial cells or from dermal fibroblasts had no major effect on DPC proliferation (S5 Fig). Importantly, direct treatment with VEGF-C did not affect DPC proliferation (Fig 4C), suggesting that VEGF-C acts via LECs to modulate HF cyling.

### LEC conditioned media promote IGF1 and ALP mRNA expression, and inhibit BMP-2, BMP-4 and TGF-β1 mRNA expression in dermal papilla cells

After incubation of DPCs with LEC-CM (30%, 50% or 90%) for 72 h, mRNA expression levels of IGF-1 were markedly enhanced (Fig 4D). Incubation with LEC-CM also potently increased the mRNA expression levels of alkaline phosphatase (ALP) that maintains the inductive properties of the dermal papilla [41, 42] (Fig 4E). This was not the case when DPCs were treated with CM from dermal blood vascular endothelial cells or from dermal fibroblasts (Fig 4D and Fig 4E). LEC-CM also increased the mRNA expression levels of FGF-10, a known promoter of hair growth [38, 43], though less potently than blood vascular endothelial cell-CM (Fig 4F). Conversely, incubation of DPCs with LEC-CM markedly decreased the mRNA expression levels of BMP-2 and BMP-4 that have been reported to inhibit HF growth [38, 44] (Fig 4G and Fig 4H). The mRNA expression levels of TGF-β1, another inhibitor of HF growth [45], were also reduced after LEC-CM incubation (Fig 4I). CM from blood vascular endothelial cells or from dermal fibroblasts had no major effects on the expression of these genes. Taken together, these results identify potential paracrine mechanisms by which lymphatic endothelium might activate DPCs.

## Discussion

In this study, we identified perifollicular LVs as new players in the coordination and regulation of HF cycling. We found that lymphatic vessels in the skin form a network that connects the individual hair follicles, and that during the anagen phase, dermal LVs reside in close proximity to murine HFs, particularly the dermal papilla and the stem cell area in the bulge. Importantly, using genetic mouse models, we demonstrate that increased dermal levels of the lymphangiogenesis factor VEGF-C lead to prolongation of anagen HF growth, whereas VEGF-C depletion resulted in accelerated catagen progression of the HFs. Similarly, exogenous administration of VEGF-C by intradermal injection accelerated the telogen-to-anagen transition.

Previously, it has been found that VEGF-A induces proliferation of DPCs through the VEGFR-2/ERK pathway and has a direct stimulatory effect on DPCs [46]. On the contrary, in this study, VEGF-C treatment did not directly increase the proliferation of DPCs. Together with the strong expansion of LVs in the skin of K14-VEGF-C transgenic mice and the spatial association of LVs with the dermal papilla during the anagen phase, our data indicate that VEGF-C might promote hair growth indirectly via a paracrine effect of VEGF-C-activated LEC on DPCs.

When cocultured with human microvascular endothelial cells, proliferation of DPCs was significantly increased [47]. Here, we found that also LEC conditioned media promoted cell proliferation of DPCs. Moreover, LEC conditioned media, in contrast to media conditioned by other relevant dermal cell populations such as blood vascular endothelial cells and dermal fibroblasts, significantly increased IGF-1, FGF-10 and ALP mRNA expression and inhibited BMP-2, BMP-4 and TGF-ß1 by DPCs, suggesting a potentially increased capacity of these cells to induce HF stem cell activation.

Beyond their physiological role in the normal hair cycle, LVs are likely involved in pathological hair loss as well, and activation of LVs might serve as a potential new treatment option for such conditions. Some reports have indicated that in permanent alopecias, such as lichen planopilaris and discoid lupus erythematosis, infiltrating immune cells surround the bulge region, whereas in reversible alopecias, such as alopecia areata, T lymphocytes directly infiltrate the bulb region [48–50]. We previously found that during chronic skin inflammation, the lymphatic vessel drainage function is impaired, associated with dilation of LVs, and that activation of LVs reduces chronic skin inflammation [9, 18, 20]. Dilated LVs were also observed in a mouse model of alopecia areata [51] and in human lipedematous alopecia [52]. It will be of interest to investigate whether activation of the lymphatic system, for example by administration of VEGF-C, might protect HFs from inflammation-associated damage.

In conclusion, these results reveal an important function of the LV network in the coordination and regulation of the HF cycle, and they indicate potential new therapeutic strategies for the treatment of conditions associated with hair loss.

## Supporting information

S1 Fig Comparable blood vessel density in WT and K14-VEGF-C mice. (TIF)

S2 Fig Increased VEGF-C mRNA expression levels during the anagen phase. (TIF)

S3 Fig VEGF-C content in DPC-CM and DF-CM. (TIF)

S4 Fig Epidermal thickness in WT and K14-VEGF-C transgenic mice is comparable. (TIF)

S5 Fig Neither DF nor BEC conditioned media do not affect DPC proliferation. (TIF)

S1 Table Sequences of primers used for human genes. (TIF)

S1 Video Ultramicroscopy image of back skin (anagen phase) stained for LYVE-1 (red) and cytokeratin 15 (green). (MOV)

S2 Video Ultramicroscopy image of back skin (telogen phase) stained for LYVE-1 (red) and cytokeratin 15 (green). (MOV)
