# Supplementary figures and images for: An important role of cutaneous lymphatic vessels in coordinating and promoting anagen hair follicle growth
Source: PLoS One. 2019 Jul 25;14(7):e0220341. doi: 10.1371/journal.pone.0220341 (PMC6657912; doi:10.1371/journal.pone.0220341)

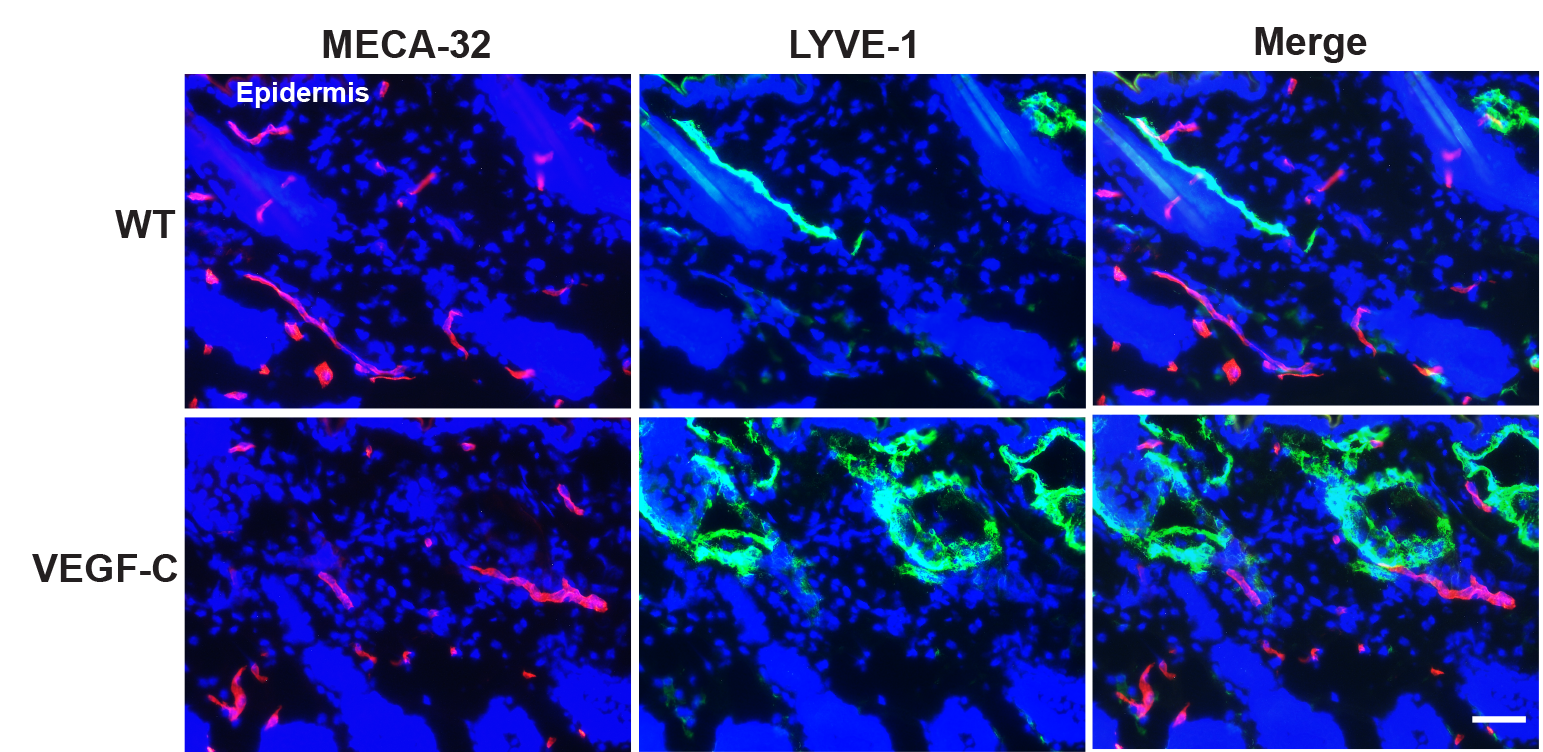

Supplement: S1 Fig — (TIF) [file pone.0220341.s001.tif]

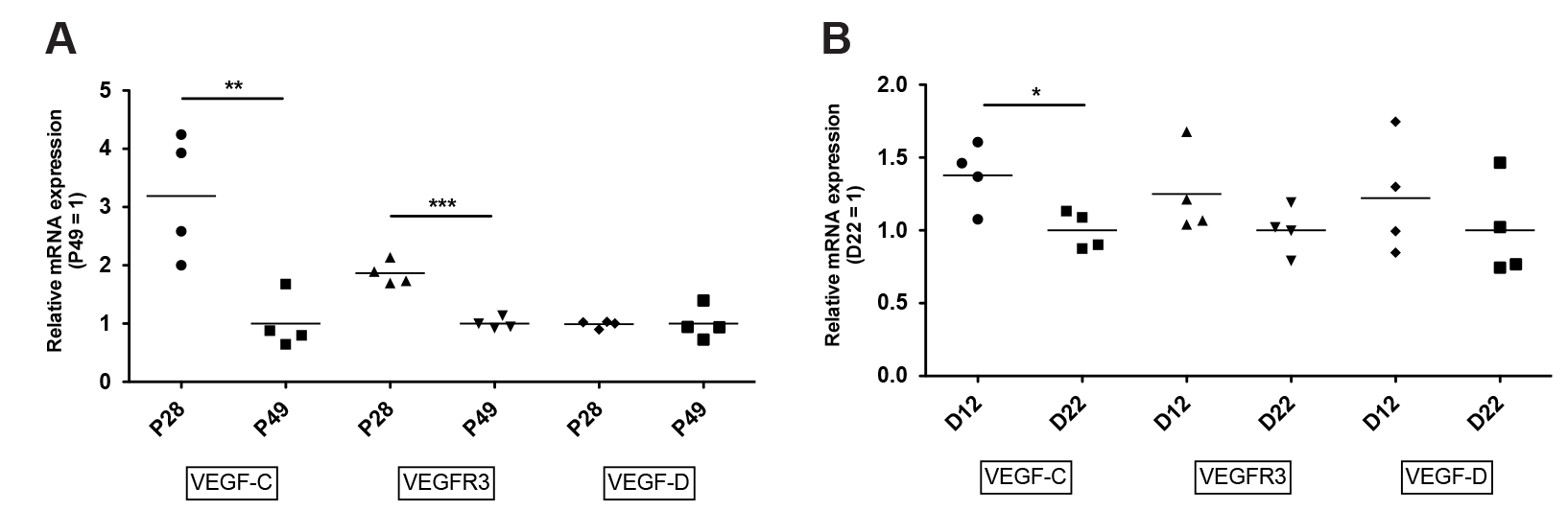

Supplement: S2 Fig — (TIF) [file pone.0220341.s002.tif]

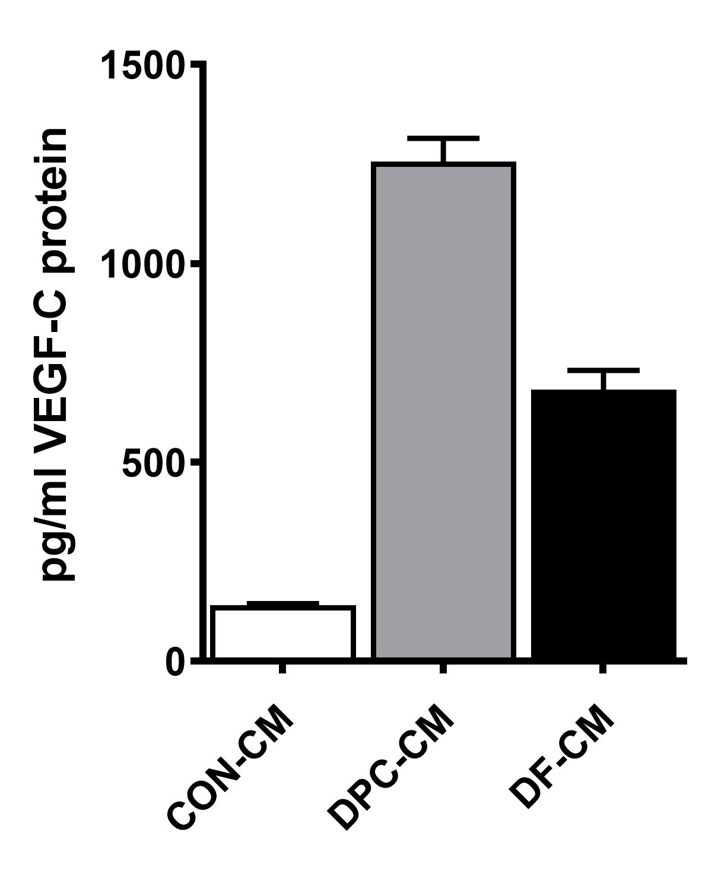

Supplement: S3 Fig — (TIF) [file pone.0220341.s003.tif]

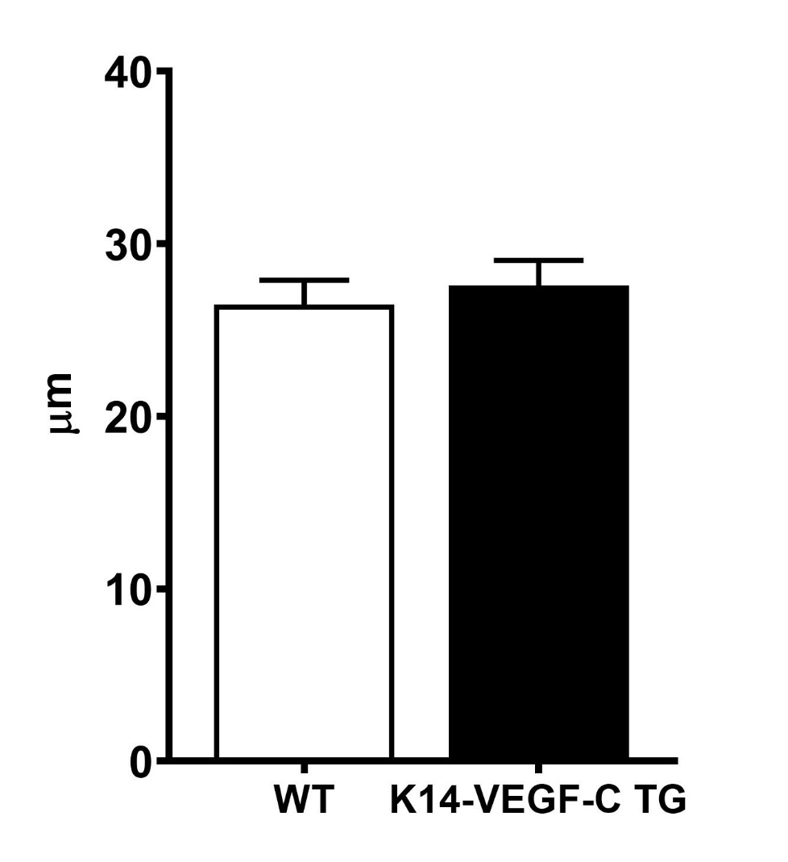

Supplement: S4 Fig — (TIF) [file pone.0220341.s004.tif]

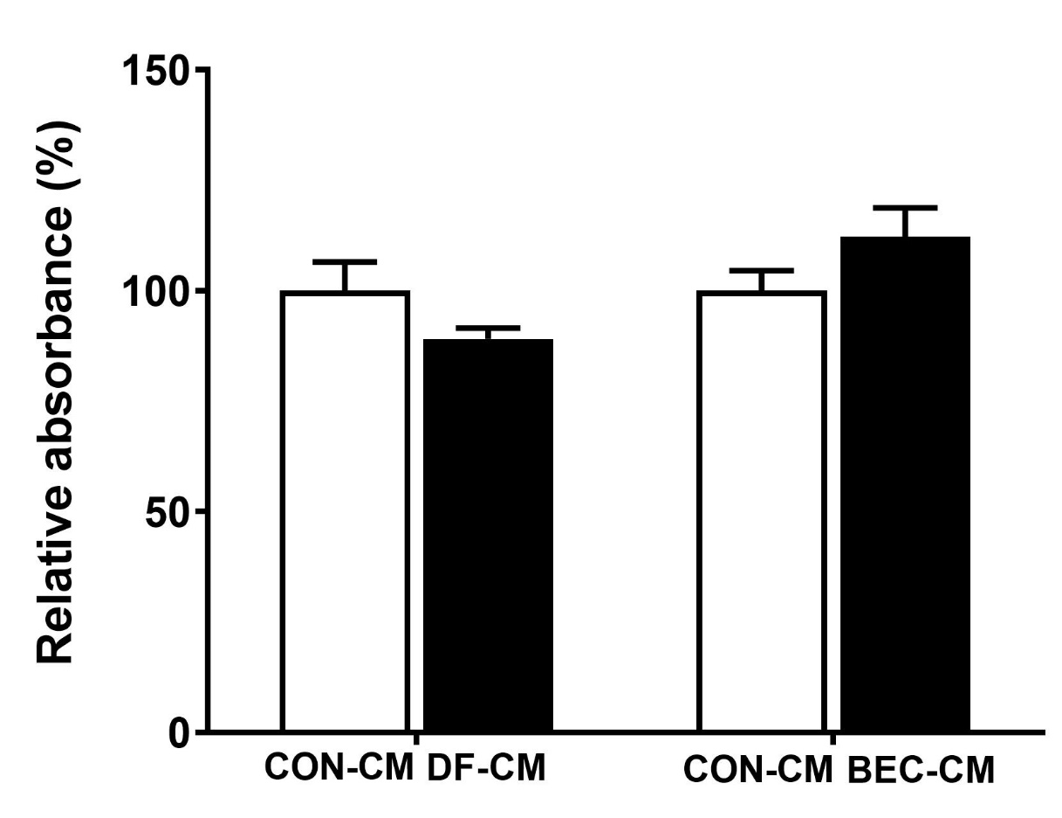

Supplement: S5 Fig — (TIF) [file pone.0220341.s005.tif]

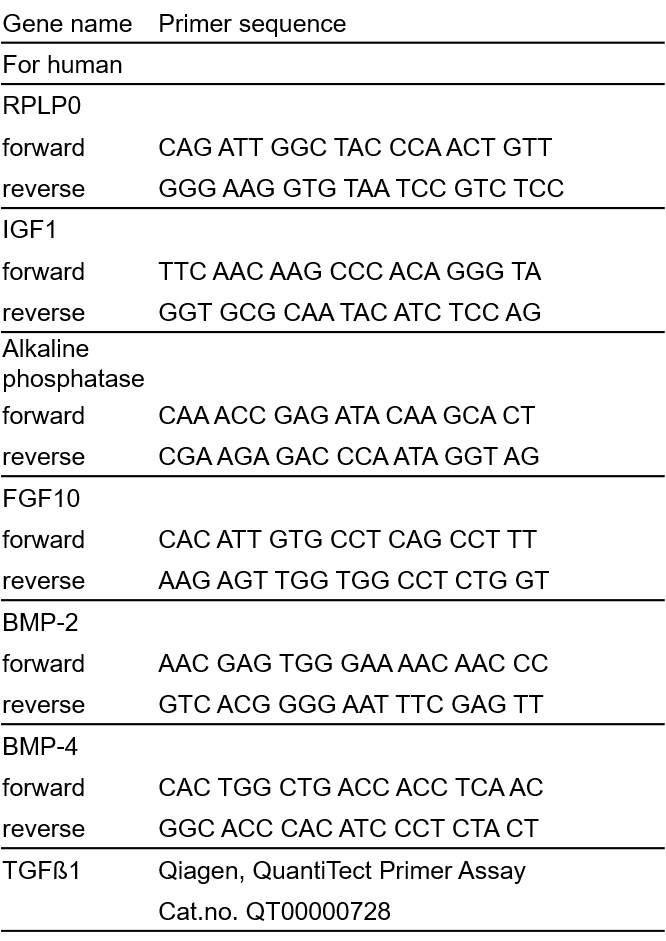

Supplement: S1 Table — (TIF) [file pone.0220341.s006.tif]
